# Multi-level Factors Impacting Implementation of Outpatient Palliative Care: Perceptions of VA Providers

**DOI:** 10.1093/geroni/igaf122.2008

**Published:** 2025-12-31

**Authors:** Brystana Kaufman, Tiera Lanford, Nina Sperber, Jessica Ma, Joshua Thorpe, Susan Hastings, David Bekelman, Courtney Van Houtven

**Affiliations:** Duke University, Durham, North Carolina, United States; Department of Veterans Affairs, Durham, North Carolina, United States; Department of Veterans Affairs, Durham, North Carolina, United States; Department of Veterans Affairs, Durham, North Carolina, United States; The University of North Carolina at Chapel Hill, Chapel Hill, North Carolina, United States; Durham VA Healthcare System, Durham, North Carolina, United States; Department of Veterans Affairs, Auroroa, Colorado, United States; Duke University, Durham, North Carolina, United States

## Abstract

Strategies to support implementation and sustainability of palliative care (PC) are needed to better meet palliative needs for older adults living in the community. Applying an implementation science framework, this qualitative study explores barriers and facilitators impacting the implementation of outpatient PC, as perceived by PC team members in the Veterans Health Administration (VHA). Provider perceptions were explored using semi-structured group interviews with 9 outpatient specialty PC teams. Participants (n = 26) included outpatient PC team members and team leaders. Barriers and facilitators were identified for key implementation and sustainability constructs within the provider, organizational context, and external levels. At the provider level, providers reported barriers including limited staffing and fragmented teams; however, they also felt that interdisciplinary and cohesive teams were facilitators to high quality PC. Teams reported that referrals they receive are not always appropriate for specialty PC, and education of referring providers was critical. At the organization level, teams embedded within a specialty or primary care clinics viewed co-location as a facilitator for referrals and care coordination. At the external environment level, most felt that traditional methods of health system performance tracking (relative value units) may undervalue the role of PC in supporting Veterans. Improved performance measures are needed to document and incentivize appropriate referral and use of PC throughout the continuum of care. Key considerations for health systems seeking to implement or expand outpatient PC include addressing misperceptions, co-location with referring clinical teams, improved performance measures, and building the PC workforce to support interdisciplinary PC teams.

